# Generation of octave-spanning mid-infrared pulses from cascaded second-order nonlinear processes in a single crystal

**DOI:** 10.1038/s41598-017-11652-9

**Published:** 2017-09-11

**Authors:** Yanchun Yin, Xiaoming Ren, Andrew Chew, Jie Li, Yang Wang, Fengjiang Zhuang, Yi Wu, Zenghu Chang

**Affiliations:** Institute for the Frontier of Attosecond Science and Technology, CREOL and Department of Physics, University of Central Florida, Orlando, Florida 32816 USA

## Abstract

We report on experimental generation of a 6.8 *μ*J laser pulse spanning from 1.8 to 4.2 *μ*m from cascaded second-order nonlinear processes in a 0.4-mm BiB_3_O_6_ (BIBO) crystal. The nonlinear processes are initiated by intra-pulse difference frequency generation (DFG) using spectrally broadened Ti:Sapphire spectrum, followed by optical parametric amplification (OPA) of the DFG pulse. The highest energy, 12.6 *μ*J, is achieved in a 0.8-mm BIBO crystal with a spectrum spanning from 1.8 to 3.5 *μ*m. Such cascaded nonlinear processes are enabled by the broadband pump and the coincident phase matching angle of DFG and OPA. The spectrum is initiated from the DFG process and is thus expected to have passive stable carrier-envelope phase, which can be used to seed either a chirped pulse amplifier (CPA) or an optical parametric chirped pulse amplifier (OPCPA) for achieving high-energy few-cycle mid-infrared pulses. Such cascaded second-order nonlinear processes can be found in many other crystals such as KTA, which can extend wavelengths further into mid-infrared. We achieved a 0.8 *μ*J laser pulse spanning from 2.2 to 5.0 *μ*m in KTA.

## Introduction

Few-cycle, mJ-level, and carrier-envelope phase (CEP) stable lasers in the short-wavelength infrared to mid-wavelength infrared are of significant interest to the ultrafast community. One of such interests is to produce isolated soft/hard X-ray attosecond pulses with shorter pulse duration and sufficient photon flux. So far, isolated extreme ultraviolet (XUV) attosecond pulses as short as 67 as have been achieved in high harmonic generation (HHG) pumped by spectrally-broadened Ti:Sapphire lasers^1^. Recent development of few-cycle and high-energy driving lasers with center wavelengths varying from 1.6 *μ*m to 2.1 *μ*m has already enabled the generation of the attosecond soft X-ray pulses in the water-window region (280 to 530 eV)^2–4^. In order to further extend the HHG cutoff photon energy and thus produce shorter attosecond pulses, the development of high-energy few-cycle pulses further into the mid-infrared (mid-IR) region is in demand because of the quadratic scaling of the ponderomotive energy with driving wavelength^5, 6^. Mid-IR laser pulses centered at 3.9 *μ*m has produced X-ray photons with more than 1.6 keV^7^. In addition, such mid-IR laser sources are ideal tools for the study of HHG in solids^8, 9^, incoherent hard X-ray generation^10^, acceleration of electrons in dielectric structures and plasma^11–13^, breakdown of dipole approximation^14^, filamentation in air^15^, rotational and vibrational dynamics of molecules^16^, time-resolved imaging of molecular structures^17^, strong-field science in plasmonic systems^18^, and mid-IR supercontinuum generation^19^.

Currently, most few-cycle long-wavelength lasers are centered around 2 *μ*m or below^20–28^, which are mainly achieved by using either optical parametric chirped pulse amplification (OPCPA) or dual-chirped optical parametric amplification (DC-OPA)^29, 30^ pumped by picosecond lasers. Most of those lasers are seeded via intra-pulse difference frequency generation (DFG)^20–22, 24, 25, 27, 28^. Intra-pulse DFG^31–33^ has several advantages: more than one octave super broad phase matching bandwidth, no jitter between the pump and the signal, and passive stable CEP for the idler since the pump and the signal are within a same pulse. In addition, it would be much desirable to obtain *μ*J-level DFG energy, which would reduce amplification stages and superfluorescence in either OPCPA or DC-OPA.

Rapid progress has been made on the generation of high-energy short-pulse mid-IR laser sources above 3 *μ*m^34–38^. However, the spectral bandwidth has not reached near one octave, which limits the pulse duration to multi-cycle. Recently, generation of octave mid-IR pulses have been theoretically investigated in mid-IR filaments^39^ and experimentally enabled with *μ*J-level energy by four-wave mixing in a two-color induced filament^40^, supercontinuum generation^19^, and adiabatic DFG^41, 42^. Especially, the adiabatic DFG^42^ has demonstrated the capabilities of octave-spanning spectrum, arbitrary pulse shaping, and compression approaching single-cycle pulse duration, although the system is complex. In this Report, we experimentally generated *μ*J-level octave-spanning mid-IR pulses with both passive-stable CEP and high efficiency by using intra-pulse DFG in a relatively simple setup. Specifically, we report on experimental generation of a 6.8 *μ*J pulse spanning from 1.8 to 4.2 *μ*m by utilizing cascaded second-order nonlinear processes^43, 44^, which are initiated by intra-pulse DFG in a 0.4-mm BiB_3_O_6_ (BIBO) crystal and simultaneously amplified in the same BIBO by optical parametric amplification (OPA). Similarly, by using a 0.8-mm BIBO crystal, we achieved a 12.6 *μ*J pulse spanning from 1.8 to 3.5 *μ*m. Such cascaded second-order nonlinear processes occur due to the coincident phase matching angle of DFG and OPA. To the best of our knowledge, this is the first time such high-energy broadband pulses around 3 *μ*m has been generated by intra-pulse DFG, which can be used to seed a two-cycle 2.4–4.0 *μ*m DC-OPA in MgO-doped LiNbO_3_ (or KNbO3)^45^, a sub-cycle 4–12 *μ*m OPCPA in ZnGeP_2_ (ZGP)^46^ or a chirped pulse amplifier with Cr^2+^:ZnSe/ZnS^47^. Moreover, we have found that such cascaded second-order nonlinear processes can occur in many other nonlinear crystals, which will open up opportunities to generate wavelength further into mid-infrared. For example, we experimentally generated a 0.8 *μ*J laser pulse spanning from 2.2 to 5.0 *μ*m in KTA by utilizing the cascaded second-order nonlinear processes. Such mid-infrared pulse can seed a Fe^2+^:ZnSe CPA laser^48, 49^.

## Results

### Experimental Setup

**Figure 1:**
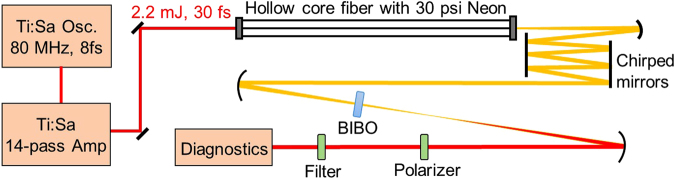
Experimental setup for the generation of multi-octave MIR spectrum via cascaded second-order nonlinear processes, i.e., DFG and OPA in a single BIBO crystal. The HCF is polyimide coated fused silica capillary tubing, which has a core size of 500 *μ*m.

The experimental setup is shown in Fig. 1. Nanojoule-level oscillator pulses (730–830 nm) are stretched to 360 ps in an Offner-type stretcher before the pulse energy is boosted by a home-made 14-pass Ti:Sapphire amplifier system to 4 mJ, out of which 2.6 mJ is compressed to 2.2 mJ, 30 fs by a transmission grating pair and is then sent to a hollow-core fiber (HCF) filled with 30 psi neon for white light generation (WLG). The white light is compressed to ~7 fs using seven pairs of chirped mirrors combined with fused silica compensating plates, after which about 700 *μ*J energy is usable for experiments. The uncoated BIBO crystal, which has a phase matching angle *θ* = 10.3° in XZ plane, was placed about 30 cm away from a f = 0.5 m concave mirror. The beam after BIBO was collimated with another f = 0.5 m concave mirror.

### Principle of cascaded second-order nonlinear processes

**Figure 2:**
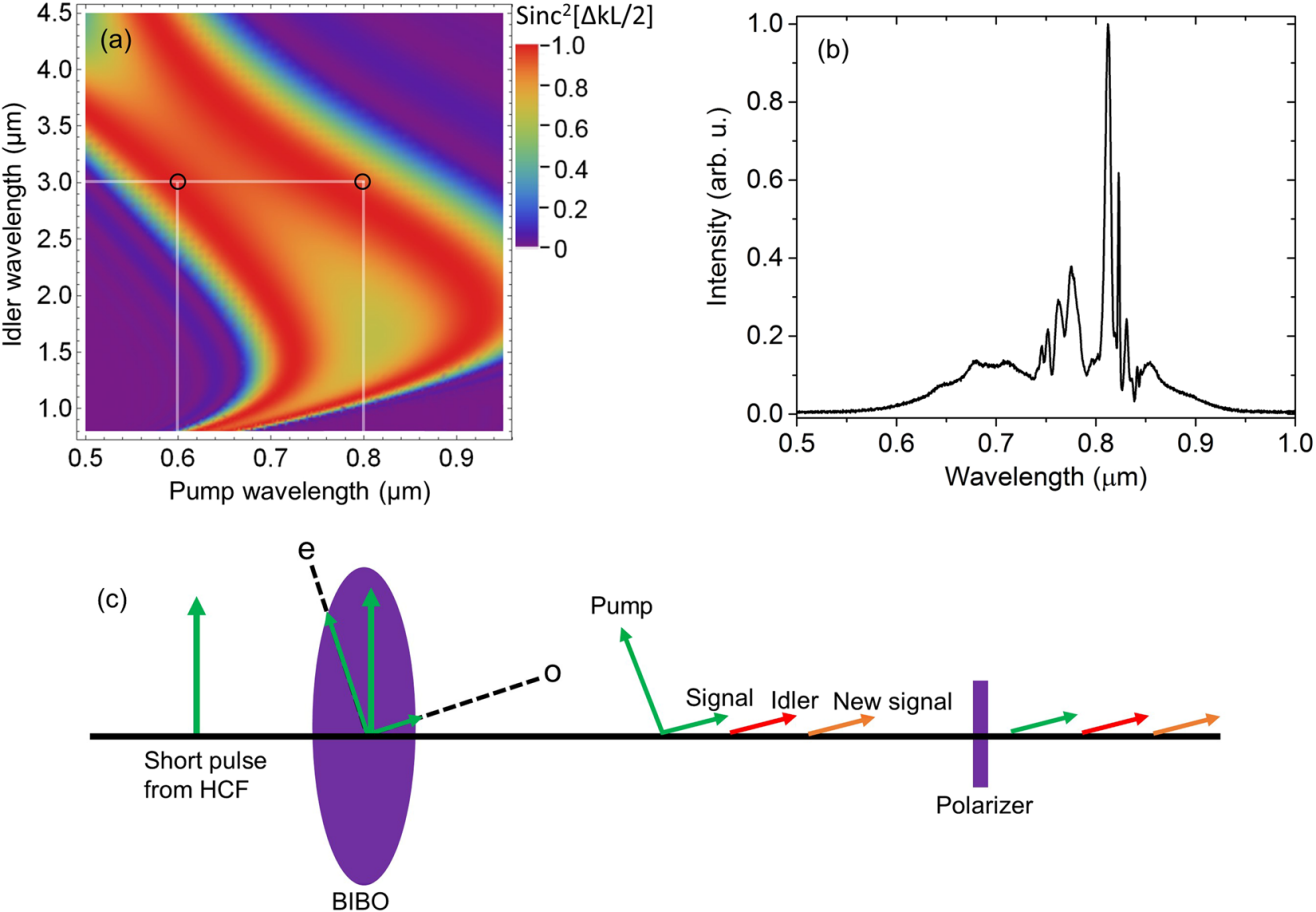
(**a**) The calculated phase matching efficiency (Sinc^2^[ΔkL/2]) as a function of pump and signal wavelengths for a 0.4 mm Type I (phase matching angle = 10.3° in the XZ plane) BIBO^50^ crystal. Δk is the propagation constant difference and L is the BIBO crystal length. The center of the left circle (black) and the right circle (black) show one example of phase matching of the DFG process and the OPA process, respectively; (**b**) the input pump spectrum generated from a broadened Ti:Sapphire spectrum in a HCF; (**c**) illustration of cascaded second-order nonlinear processes in the type I phase matching of BIBO, where a major part of HCF pulses is along the e axis of the BIBO crystal and a small portion is along the o axis.

The principle of cascaded second-order nonlinear processes is illustrated in Fig. 2. Figure 2(a) shows the normalized phase matching efficiency of a 0.4-mm BIBO, where second-order nonlinear processes occurring at different wavelengths can be conveniently located. The first second-order nonlinear process–intra-pulse DFG–is within WLG pulses. The pump is from the short wavelengths and the signal is from the long wavelengths of the WLG spectrum. The input WLG pulse is linearly polarized. If the WLG pulse polarization is purely along either the ordinary (o) axis or the extra-ordinary (e) axis of the BIBO crystal, there is no phase matched second-order nonlinear process. In order to initiate the DFG process efficiently, the BIBO crystal has to be orientated in such a way that a major portion of the WLG pulse energy is projected along the e axis and a small portion is projected along the o axis, as shown in Fig. 2(c). One example of phase matching in DFG, which is illustrated by the center of the left circle in Fig. 2(a), is shown as follows:

1 $$\hslash {\omega }_{pump\mathrm{:600}nm}(e)-\hslash {\omega }_{signal\mathrm{:750}nm}(o)=\hslash {\omega }_{idler\mathrm{:3000}nm}(o)$$

Once DFG is initiated, the long wavelengths of the WLG pulse will serve as a pump and the DFG pulse will serve as an idler in the second second-order nonlinear process–OPA, in which a new signal around and above 1 *μ*m will be generated and in the meantime, the DFG pulse will be amplified. One example of such phase matching in the OPA, which is illustrated by the center of the right circle in Fig. 2(a), is shown as follows:

2 $$\hslash {\omega }_{pump\mathrm{:800}nm}(e)-\hslash {\omega }_{idler\mathrm{:3000}nm}(o)=\hslash {\omega }_{signal\mathrm{:1091}nm}(o)$$

Thus, the two second-order nonlinear processes share a same idler, which is initiated by the DFG process and amplified by the OPA process. This idler is expected to be CEP stable and is the focus of this Report. From the phase matching plot such as the one in Fig. 2, one can obtain all the phase-matching data for predicting the cascaded processes. The first step is to identify available pump wavelengths (HCF spectrum in the case of this manuscript). With given pump wavelengths, all the idler wavelengths that are within the phase matching region can be located. Finally, all the signal wavelengths can be calculated from the pump and idler wavelengths.

### Experimental results

In the experiments, to optimize the conversion efficiency from pump to idler in the two combined second-order nonlinear processes, the angle between the input WLG pulse polarization and the e axis of the BIBO was set to be 8°. About 98% of the WLG pulse energy was along the e axis of the BIBO and the remainder was along the o axis. After the cascaded nonlinear processes, a wire polarizer was used to block the e polarization and only allows the o polarization to pass through. A 69 *μ*J spectral continuum in the o polarization spanning from 0.5 *μ*m to 4.2 *μ*m was obtained in a 0.4-mm BIBO and a 98 *μ*J continuum spanning from 0.5 *μ*m to 3.5 *μ*m was obtained in a 0.8-mm BIBO. Four spectrometers were used for the measurements of spectra: a 0.5–0.95 *μ*m NIR spectrometer, a 0.9–1.3 *μ*m InGaAs spectrometer, a 1.1–2.5 *μ*m IR spectrometer, and a 1.5–6.3 *μ*m spectrometer. The spectra from different spectrometers were stitched together while taking into account of the relative spectral intensity.

**Figure 3:**
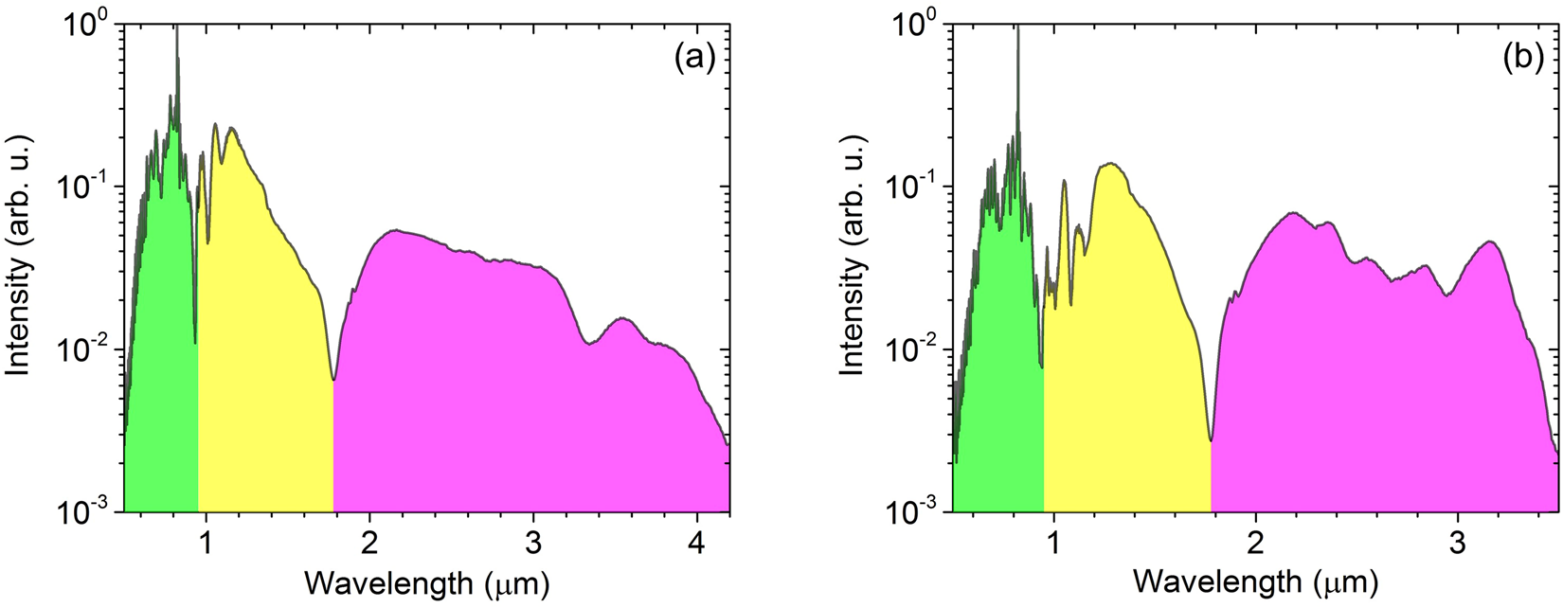
Measured output spectrum from (**a**) 0.4 mm and (**b**) 0.8 mm BIBO crystals with the polarization parallel to the o axis of BIBO crystals, including a small portion of HCF spectrum (0.5–0.95 *μ*m), the idler spectrum (1.8–4.2 *μ*m & 1.8–3.5 *μ*m) from DFG, and the new signal spectrum (0.95–1.8 *μ*m) generated in the OPA process when the DFG pulse is amplified.

The measured two continuum spectra were shown in Fig. 3. In the continuum, the 0.5–0.95 *μ*m spectrum was from the original WLG pulse, which was involved in the DFG process. The 0.95–1.8 *μ*m spectrum was the signal generated from the second nonlinear process–OPA. The 1.8–4.2 *μ*m spectrum in Fig. 3(a) or the 1.8–3.5 *μ*m spectrum in Fig. 3(b) was from the DFG process, which was further amplified by the OPA process. The reduced bandwidth in the 0.8-mm BIBO crystal is due to increased group velocity mismatch during the nonlinear processes. Although it is known that BIBO has a limited transparency at around 3 *μ*m, the idler generated in BIBO can reach 4.2 *μ*m due to the very thin BIBO–0.4 mm. Nevertheless, the obtainable idler bandwidth in the 0.4 mm BIBO is still limited by optical absorption as the theoretical phase matching can go beyond 4.5 *μ*m. Thus, the idler bandwidth is limited by optical absorption in a relatively thin BIBO crystal and is limited by phase matching in a relatively thick BIBO crystal.

**Figure 4:**
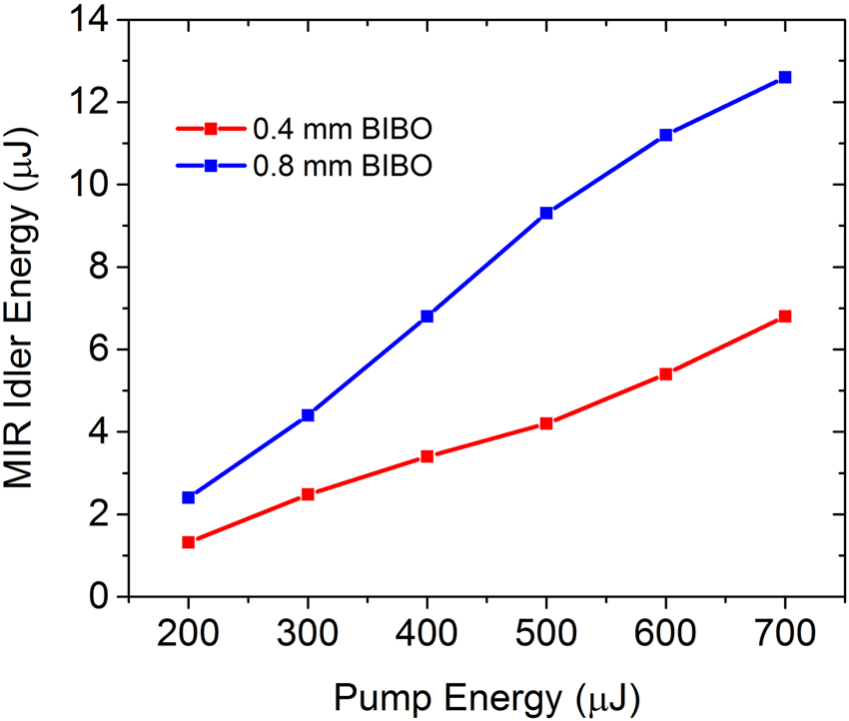
Measured idler energy as a function of input pump energy in 0.4 mm (red) and 0.8 mm (blue) BIBO crystals.

**Figure 5:**
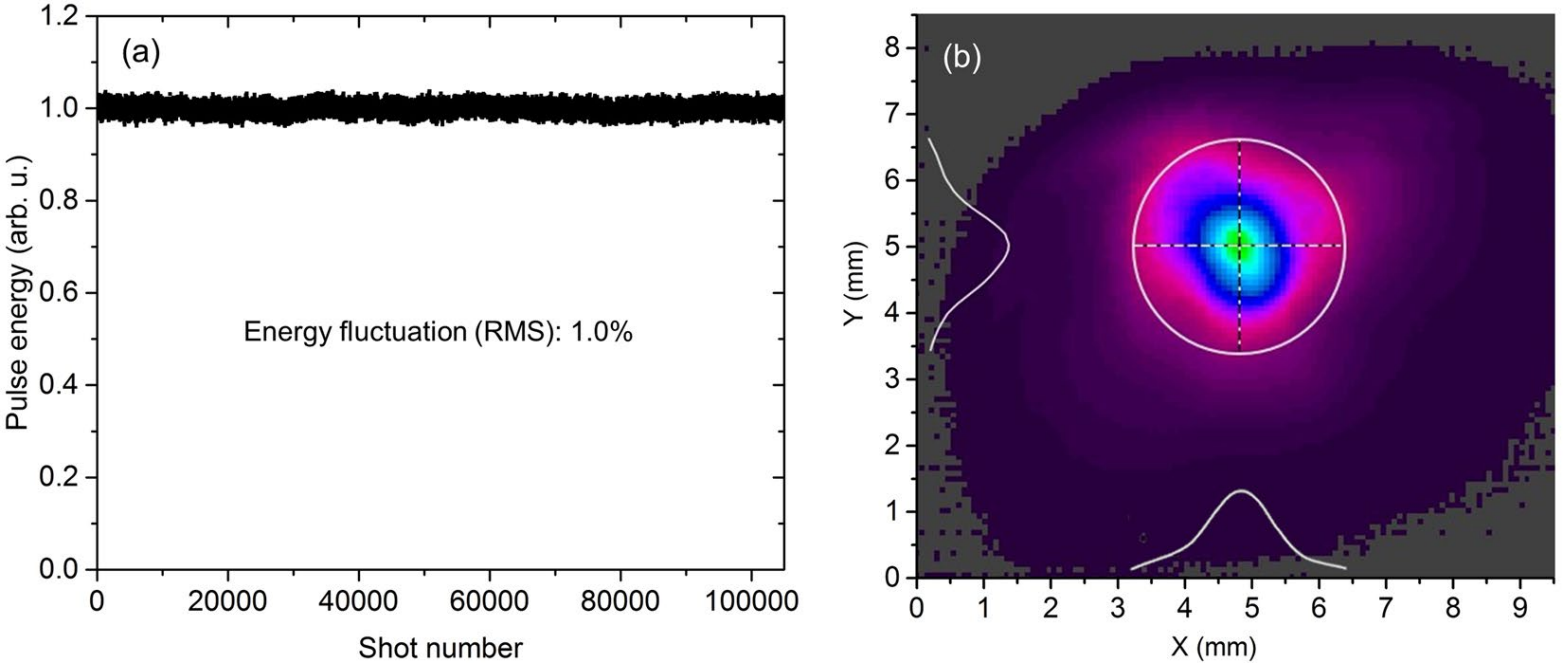
(**a**) Idler pulse energy stability; (**b**) idler beam profile.

Since we are primarily interested in the spectral region (>1.8 *μ*m) originated from DFG, the energy scaling for the idler was studied, which is shown in Fig. 4. A long-pass filter was used to block the spectrum below 1.8 *μ*m. The output idler pulse energy changes linearly with the input WLG pulse energy in both a 0.4-mm and a 0.8-mm BIBO crystal. A 6.8 *μ*J pulse covering 1.8–4.2 *μ*m was obtained in a 0.4-mm crystal, compared with a 12.6 *μ*J pulse covering 1.8–3.5 *μ*m in a 0.8-mm BIBO crystal. Further energy scaling in both crystals is limited by the damage threshold of BIBO with currently available 700 *μ*J input WLG pulse energy. The pulse energy fluctuation (RMS) was about 1.0% measured over 100,000 laser shots, as shown in Fig. 5(a). The idler beam has a near Gaussian spatial profile, as shown in Fig. 5(b).

## Discussion

Such cascaded nonlinear processes also exist in many other nonlinear crystals. By replacing the BIBO crystal, we have found experimentally that a 0.2-mm KTA can generate a 0.8 *μ*J pulse covering 2.2–5.0 *μ*m when pumped with a 700 *μ*J compressed WLG pulse generated in HCF. The KTA crystal is uncoated and has a phase matching angle *θ* = 41° in the XZ plane.

**Figure 6:**
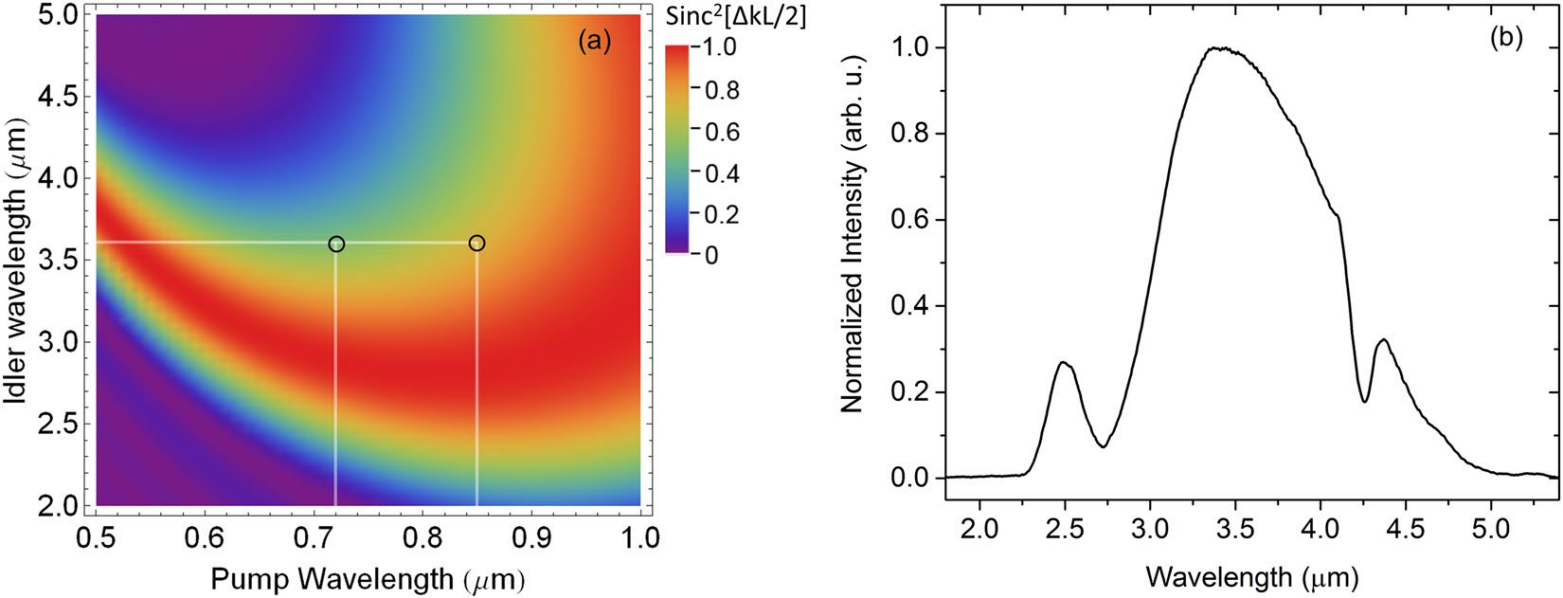
(**a**) The calculated phase matching efficiency (Sinc^2^[ΔkL/2]) as a function of pump and signal wavelengths for a 0.2-mm Type I (phase matching angle = 41° in the XZ plane) KTA^52^ crystal. Δk is the propagation constant difference and L is the KTA crystal length. The center of the left circle (black) and the right circle (black) show one example of phase matching of the DFG process and the OPA process, respectively; (**b**) the idler spectrum generated by the cascaded second-order nonlinear processes in KTA.

The principle of cascaded second-order nonlinear processes in KTA is illustrated by the phase matching curve shown in Fig. 6(a). One example of phase matching in DFG, which is illustrated by the center of the left circle in Fig. 6(a), is shown as follows:

3 $$\hslash {\omega }_{pump\mathrm{:720}nm}(e)-\hslash {\omega }_{signal\mathrm{:900}nm}(o)=\hslash {\omega }_{idler\mathrm{:3600}nm}(o)$$

One example of phase matching for amplifying DFG in the OPA, which is illustrated by the center of the right circle in Fig. 2(a), is shown as follows:

4 $$\hslash {\omega }_{pump\mathrm{:850}nm}(o)-\hslash {\omega }_{idler\mathrm{:3600}nm}(o)=\hslash {\omega }_{signal\mathrm{:1113}nm}(e)$$

It is worth noticing that the pump (e) for DFG and the pump (o) for OPA have orthogonal polarization, which is unlike the case in BIBO where both pumps are along the e axis. In order to optimize the conversion efficiency of the cascaded processes, the angle between the input WLG pulse polarization and the e axis of the KTA was set to be about 45°. Thus, the pump energy was almost equally shared between the DFG process and OPA process, which in part explains the fact that the efficiency of cascaded processes in KTA is not as efficient as that in BIBO. Further energy scaling in a longer KTA crystal would be expected with sacrifice of reduced bandwidth. Nevertheless, this 2.2–5.0 *μ*m broadband pulse can seed a Fe^2+^:ZnSe CPA laser^48, 49, 51^.

Other crystals that allow for cascaded nonlinear processes include KNbO_3_, LiIO_3_, and LiNbO_3_, among which KNbO_3_ is the best candidate due to its large second-order nonlinearity and high damage threshold. The nonlinear processes in those crystals are not as efficient as in BIBO, but can extend DFG wavelengths beyond 5 *μ*m.

Although the duration is unknown, it cannot be very long, given the short length of the conversion crystal. We are not concerned with the idler pulse duration here since the idler would be primarily used to seed either a Cr^2+^:ZnSe CPA^47^ or an OPCPA^45, 46^, and the amplified pulse can be compressed by using an acousto-optic programmable dispersive filter (AOPDF) combined with a grating pair or a bulk material.

## Conclusions

In conclusion, we have experimentally generated a 6 *μ*J laser pulse spanning from 1.8 to 4.2 *μ*m in a 0.4-mm BIBO and a 12.6 *μ*J laser pulse spanning from 1.8 to 3.5 *μ*m in a 0.8-mm BIBO by utilizing cascaded second-order nonlinear processes. The mid-IR idler pulse above 1.8 *μ*m is generated in the intra-pulse DFG process and is further amplified by a second second-order nonlinear process–OPA. We also experimentally demonstrated that the cascaded second-order nonlinear processes in KTA can produce a 0.8 *μ*J pulse with a spectrum spanning from 2.2 to 5.0 *μ*m. The mid-IR pulse generated and amplified by the cascaded processes is expected to have passive stable CEP, which can be used to seed either a CPA or an OPCPA for achieving high-energy few-cycle mid-infrared pulses. Besides, such cascaded nonlinear processes in crystals such as KNbO_3_ may pave the way for high-energy seeding at even longer mid-infrared wavelengths.
